# Extracellular vesicles‐derived miR‐150‐5p secreted by adipose‐derived mesenchymal stem cells inhibits CXCL1 expression to attenuate hepatic fibrosis

**DOI:** 10.1111/jcmm.16119

**Published:** 2020-12-20

**Authors:** Zhiyong Du, Tianchong Wu, Linsen Liu, Biwei Luo, Cuifeng Wei

**Affiliations:** ^1^ Department of Hepatobiliary and Pancreatic Surgery Shenzhen People’s Hospital (The Second Clinical Medical College Jinan University Guangzhou China; ^2^ The First Affiliated Hospital Southern University of Science and Technology) Shenzhen China; ^3^ Department of Endocrinology Jingmen First People’s Hospital Jingmen China

**Keywords:** adipose‐derived mesenchymal stem cells, extracellular vesicles, hepatic fibrosis, hepatic stellate cells, MicroRNA‐150‐5p

## Abstract

Hepatic fibrosis (HF) is involved in aggravated wound‐healing response as chronic liver injury. Extracellular vesicles (EVs) carrying microRNA (miR) have been reported as therapeutic targets for liver diseases. In this study, we set out to explore whether adipose‐derived mesenchymal stem cells (ADMSCs)‐derived EVs containing miR‐150‐5p affect the progression of HF. Carbon tetrachloride (CCl_4_) was firstly used to induce HF mouse models in C57BL/6J mice, and activation of hepatic stellate cells (HSCs) was achieved using transforming growth factor β (TGF‐β). EVs were then isolated from ADMSCs and co‐cultured with HSCs. The relationship between miR‐150‐5p and CXCL1 was identified using dual luciferase gene reporter assay. Following loss‐ and gain‐function experimentation, HSC proliferation was examined by MTT assay, and levels of fibrosis‐, HSC activation‐ and apoptosis‐related genes were determined in vitro. Additionally, pathological scores, collagen volume fraction **(**CVF) as well as levels of inflammation‐ and hepatic injury‐associated genes were determined in in vivo. Down‐regulated miR‐150‐5p and elevated CXCL1 expression levels were detected in HF tissues. ADMSCs‐derived EVs transferred miR‐150‐5p to HSCs. CXCL1 was further verified as the downstream target gene of miR‐150‐5p. Moreover, ADMSCs‐EVs containing miR‐150‐5p markedly inhibited HSC proliferation and activation in vitro. Meanwhile, in vivo experiments also concurred with the aforementioned results as demonstrated by inhibited CVF, reduced inflammatory factor levels and hepatic injury‐associated indicators. Both experiments results were could be reversed by CXCL1 over‐expression. Collectively, our findings indicate that ADMSCs‐derived EVs containing miR‐150‐5p attenuate HF by inhibiting the CXCL1 expression.

## 1 INTRODUCTION

Hepatic fibrosis (HF), a resultant of chronic liver injury, is associated with an aggravated wound‐healing response.^1^, ^2^ HF presents a rising tendency on a globe scale, because of drinking, obesity, as well as chronic hepatitis B and C.^3^ HF can often lead to severe outcomes such as liver cirrhosis or even hepatocellular carcinoma which further burden the medical infrastructure.^4^ Clinically, HF is characterized by hepatic stellate cells (HSCs) activation along with extracellular matrix accumulation.^5^ Furthermore, a diagnosis of HF can only be made with invasive procedures like biopsy, which always remain unfavourable among patients.^6^ Currently, liver transplantation is regarded as the most optimal treatment strategy for patients with end‐stage HF, but faces many obstacles such as shortage of donor organs and complications during surgery.^7^ Recently, HSCs‐specific strategy has been highlighted to possess therapeutical potential for HF therapy,^8^ while the therapeutic role of extracellular vesicles (EVs) in HF has also garnered the interest of numerous researchers.^9^ Therefore, extensive research on the effect of EVs in HF aimed at HSC activation could prove beneficial in regard to HF treatment strategies.

EVs, including exosomes and microvesicles, are known as membrane‐bound particles secreted by multiple cell types, which exert crucial functions in cell‐to‐cell communication.^10^ Meanwhile, mesenchymal stem cells (MSCs) are also known to yield paracrine functions through the release of EVs containing microRNA, mRNA, as well as proteins.^11^ Importantly, MSCs or MSC‐derived microvesicles are capable of treating hepatic diseases.^12^ More notably, adipose‐derived mesenchymal stem cells (ADMSCs) were recently highlighted to possess the ability to suppress HSC activation and attenuate HF.^13^ EVs are also capable of carrying biologically active cargos to target cells from donor cells.^14^ Additionally, microRNAs (miRs) are regarded as the cargos which can be encapsulated in EVs and transferred between cells.^15^ Recent studies have further reported that EVs secreted by ADMSCs can deliver miRs such as miR‐181‐5p and miR‐122 to inhibit the development of HF.^16^, ^17^ Interestingly, researchers have also found that one of the miR members, miR‐150, could inhibit HSC activation.^18^ Moreover, hsa‐miR‐150‐5p was previously suggested to play an important role in HF by regulating processes such as metabolism and extracellular matrix protein organization.^19^ Therefore, we have suggested that ADMSCs‐derived EVs containing miR‐150‐5p may be conducive for HF amelioration. Of note, with miR‐150‐50 as a focus of this study, our targeting binding prediction further verified CXC chemokine‐ligand‐1 (CXCL1) as the downstream target of miR‐150‐5p. In addition, a negative correlation was observed between miR‐150 and CXCL1 expression in mice overexpressing or underexpressing Kruppel‐like factor 2.^20^ CXCL1, a ligand for CXC chemokine receptor 2, has also been documented to be expressed in HSCs.^21^ In addition, CXCL1 was previously indicated as a profibrotic chemokine partially responsible for fibroinflammatory liver injuries.^22^ Taken all the above into consideration, we set out to perform a series of experiments to verify our hypothesis that miR‐150‐5p‐containing EVs derived from ADMSCs can influence the development of HF by targeting CXCL1.

## 2 MATERIALS AND METHODS

### 2.1 Ethical approval

All animal studies were approved by the Ethics Committee of Jingmen First People's Hospital and performed strictly following the Guide for Institutional Animal Care and Use Committee of Jingmen First People's Hospital. Extensive efforts were made to minimize the suffering of the included animals.

### 2.2 Experimental animals and model establishment

C57BL/6J male mice (aged 6 weeks old; calculated mean weight of 20 ± 2 g) were reared in a specific pathogen‐free grade animal laboratory. The mice were used for experimentation after two weeks of adaptive feeding, with the mice being 8 weeks old. As a result, HF mouse models were established by injecting 10 mL/kg 10% carbon tetrachloride (CCl_4_; 48 604, Sigma‐Aldrich, St. Louis, MO, USA) into the mice peritoneum twice a week, for continuous eight weeks.

### 2.3 Culture of ADMSCs

The abdominal adipose tissues were extracted after the mice were euthanized by cervical dislocation and then rinsed repeatedly with pre‐cooled phosphate‐buffered saline (PBS) containing 2% penicillin/streptomycin (15140‐122; Gibco, Carlsbad, California, USA). The isolated adipose tissues were then sliced into pieces (1 mm^3^), treated with 0.1% (mg/mL) type I collagenase digestion solution (5135; Sigma‐Aldrich, St. Louis, MO, USA) and placed in a 15‐mL centrifuge tube for detachment at 37℃ for 0.5‐1 hour. The detachment was stopped with the addition of equal volumes of basic medium (MEL08‐500ML; AmyJet Scientific, Wuhan, China). Centrifugation was subsequently conducted at room temperature at 1000 r/minute (radius of 8 cm) for 8 minutes, and the supernatant was removed. The obtained cells were then resuspended in the basic culture medium, filtered with a 200‐mesh cell sieve (DE2007; Biodee, Beijing, China) and centrifuged another time. The supernatant and suspended adipocytes were discarded before the cells were resuspended with the MSC culture medium (S1569; Sigma). The cells were seeded at a density of 5 × 10^5^ cells per 3.5‐cm culture dish and recorded as primary cells (P0); they were sub‐cultured until 80‐90% confluency in a humidified incubator with 5% CO_2_ at 37°C. The ADMSCs were subjected to sorting analysis by fluorescence activation using a BD LSRII analytical device (BD Biosciences, Franklin Lakes, NJ, USA). No spontaneous differentiation was observed during the culture process. In accordance with the instructions of the three‐line induction and differentiation kit (CHEM‐200004/5/6, Linmeng Biotechnology Co., Ltd., Shanghai, China), Alizarin Red S, oil red O and Alcian blue staining were adopted to observe the osteogenic, adipogenic and chondrogenic differentiation abilities of ADMSCs, respectively. The EV‐free serum was purchased from Shunran Biotechnology Co., Ltd. (EXO‐FBS‐50A‐1‐SBI; Shanghai, China).

Primary mouse HSCs were obtained using pronase/collagenase perfusion through gradient centrifugation. The purity of isolated HSCs was evaluated with the help of alpha smooth muscle actin (α‐SMA) staining based on immunocytochemistry with purity over 95%. Mouse HSCs were obtained by collagenase and cultured in Dulbecco modified eagle's medium (DMEM) comprising of 10% foetal bovine serum (FBS), streptomycin (100 g/mL) and penicillin (100 U/mL). The obtained cells were placed in a humidified incubator with 5% CO_2_ at 37°C.

### 2.4 Lentivirus transduction

Lentivirus packaging kits and lentiviral plasmids containing miR‐150‐5p, miR‐negative control (NC), over‐expression (oe)‐CXCL1 and oe‐NC were procured from GeneCopeia (Rockville, MD, USA). Forty‐eight hours later, a p24 ELISA kit (Cell Biolabs, Inc, San Diego, USA) was adopted to determine the virus titre (5 × 10^8^ Tu/mL for miR‐150‐5p, oe‐CXCL1; 8 × 10^8^ Tu/mL for miR‐NC, oe‐NC). Subsequently, the prepared lentiviral particles were used to infect the ADMSCs and their control cells for 24 hours. After 48 hours of culture, purinomycin (p8230, Solarbio Technology Co., Ltd., Beijing, China) was adopted to screen the stably infected cell lines.

### 2.5 Isolation and identification of EVs

The supernatant of ADMSCs (500 g) was collected and centrifuged, filtered through a 0.22 μM filter and centrifuged at 110 000 × g for 70 minutes. The precipitate was collected, resuspended with PBS, centrifuged at 110 000 × g for 70 minutes and then resuspended with 100 μL sterile PBS. All the ultracentrifugation steps were carried out at 4℃, using a Beckman ultracentrifuger (TL‐100, Beckman Coulter Inc, Chaska, MN, USA) and a TLS‐55 swing bucket rotor. Low‐speed centrifugation was performed with a Beckman Allegra X‐15R desktop centrifuge.

A total of 20 μL of EVs was dropped on a copper net and then soaked for 3 minutes. Filter paper was then used to absorb the liquid from the side, followed by the addition of 30 μL phosphotungstic acid solution (pH 6.8). EVs were subsequently counterstained at room temperature for 5 minutes, dried with an incandescent lamp and photographed under a transmission electron microscope. Particle size analysis^23^ was performed with nanoparticle tracking analysis (NS300, Malvern Instruments Ltd., Worcestershire, UK). Western blot assay was also applied to identify the surface markers of EVs. The EV suspensions were determined using a bicinchoninic acid kit (BCA; 23227, Thermo Fisher Scientific Inc, Waltham, Massachusetts, USA). After the sulphate‐polyacrylamide gel electrophoresis gel was prepared, the proteins were denatured and underwent electrophoresis. Afterwards, the proteins were transferred onto a membrane and the specific marker proteins of EVs, including tumour susceptibility gene 101 (TSG101) (ab30871, dilution ratio of 1:1000), CD63 (ab68418, dilution ratio of 1:1000) and CD81 (ab109201, dilution ratio of 1:2000) and negative control GRP94 (ab3674, dilution ratio of 1:3000) were determined. Ponceau red served as the loading control. All the aforementioned antibodies were obtained from Abcam Inc (Cambridge, MA, USA).

### 2.6 Uptake test of EVs

The purified EVs secreted by ADMSCs from normal mice (ADMSCs‐miR‐150‐5p‐EVs) were labelled using a PKH67 green fluorescence kit (PKH67GL‐1KT, Sigma). The EVs were then resuspended in 1 mL Diluent C solution, and 4 μL PKH67 ethanol dye solution was added to 1 mL Diluent C to prepare a 4 × 10^‐6^ M dye solution. Next, 1 mL EVs suspension was mixed with the dye solution for 5 minutes and incubated with 2 mL of 1% bovine serum albumin (BSA) for 1 minute to stop dyeing. The labelled EVs were subsequently ultracentrifuged at 100 000 × g for 70 minutes, ultracentrifuged again and then resuspended in 50 μL PBS. PKH67‐labelled EVs were incubated with HSCs at 37℃ for 12 hours. The cells were fixed with 4% polyformaldehyde, rinsed with PBS and the nuclei were stained using 4′,6‐diamino‐2‐phenylindole (DAPI). HSCs were co‐cultured with ADMSC‐EVs overexpressing miR‐150‐5p, and the HSCs + ADMSCs +miR‐150‐5p co‐culture system was treated with EVs inhibitor GW48699 (N,N‐Bis[4‐(4,5‐dihydro‐1H‐imidazol‐2‐yl)phenyl]‐3,3‐p‐phenylene‐bis‐acrylamide dihydrochloride, Sigma‐Aldrich, St. Louis, USA) with PBS as the control. The uptake of labelled EVs by HSCs was measured with the help of confocal microscopy (Zeiss LSM 800, Carl Zeiss, Jena, Germany).

Adipose‐derived mesenchymal stem cells were transfected with ADMSCs‐EVs containing Cy3‐miR‐150‐5p mimic (GenePharma, Shanghai, China) using a lipo3000 kit (L3000001, Invitrogen Inc, Carlsbad, CA, USA). After transfection for 6 hours, ADMSCs were cultured with 10% EVs‐free serum medium for 48 hours. The uptake of EVs (red fluorescence) with Cy3‐miR‐150‐5p by HSCs (green fluorescence) was measured using microscopy or confocal microscopy (LSM710, Carl Zeiss).

### 2.7 Immunofluorescence

After conventional detachment and transfection, the cells were counted and cultured in an immunofluorescence chamber at a density of 2 × 10^5^ cells per well. Next, 1 mL of 4% paraformaldehyde was added to fix the cells, followed by reaction with 0.3% Triton. The PBS‐prepared primary antibodies to vimentin (ab92547, dilution ratio of 1:300) and α‐SMA (ab108424, dilution ratio of 1:500) were incubated with the cells, followed by further incubation with green fluorescence‐labelled Annexin V‐fluorescein isothiocyanate (FITC)‐goat anti‐rabbit secondary antibody (ab6717, dilution ratio of 1:2000) in the dark for 1 hour at room temperature. After the nuclei were stained with DAPI in the dark for 15 minutes, the cells were observed and photographed under the same exposure condition using a fluorescent microscope.

### 2.8 3‐[4,5‐dimethylthiazol‐2‐yl]‐2,5‐diphenyltetrazolium bromide (MTT) assay

The 96‐well plates were seeded with 5 × 10^6^ ~ 6 × 10^6^ cells per well. The volume of each well was set to 0.2 mL, with six duplicate wells. The plates were taken out after 24 hours, 48 hours and 72 hours of incubation, respectively. The medium containing 10% MTT solution (5 g/L) (GD‐Y1317; Guduo Biotechnology, Shanghai, China) was then adopted for further 4 hours of culture. The supernatant was subsequently removed, and 100 μL dimethyl sulfoxide (DMSO) was added to each well (D5879‐100 mL; Sigma) to fully dissolve the crystal of methylzan produced by the living cells. Afterwards, the optical density (OD) value of each well was measured at a wavelength of 490 nm using a microplate reader (BS‐1101; Detie Experimental Equipment Co., Ltd., Nanjing, China).

### 2.9 Western blot assay

The radioimmunoprecipitation assay lysate pre‐cooled at 4℃ containing phenylmethyl sulfonyl fluoride (R0010, Solarbio, Beijing, China) was adopted to extract the total protein content from the cells or tissues. A BCA kit (20201ES76, Yeasen) was then used to determine the protein concentration of each sample. After sulphate‐polyacrylamide gel electrophoresis, the proteins were transferred onto a polyvinylidene fluoride membrane (Millipore, Bedford, MA, USA) by the wet transfer method and sealed with 5% BSA at room temperature for 1 hour. The membrane was then probed with the diluted primary rabbit antibodies to CXCL1 (3 629 493, 1:800, MGI), Bcl‐2‐associated X protein (Bax; ab32503, 1:2000), B‐cell lymphoma 2 (Bcl‐2; ab59348, 1:1000), cleaved caspase‐3 (ab49822, 1:500), collagen Ⅰ (ab34710, 1:2000), collagen Ⅲ (ab7778, 1:5000), fibronectin (ab2413, 1:2000) and glyceraldehyde‐3‐phosphate dehydrogenase (GAPDH) (ab8245, 1:5000) at 4°C. The following day, the membrane was rinsed with Tris‐buffered saline containing Tween‐20 and re‐probed with the diluent of goat anti‐rabbit to immunoglobulin G (IgG; ab6721, 1:5000) labelled with horseradish peroxidase for 1 hour at room temperature (all the above antibodies were purchased from Abcam, except CXCL1). The substrate enhanced chemiluminescence reagent of horseradish peroxidase was purchased from Lianshuo Biotechnology (WBKLS0050, Shanghai, China). Finally, with GAPDH serving as the internal reference, a gel imaging analysis system (GIS‐500, Qian Ming Gene Technology Co., Ltd., Beijing, China) and the ImageJ software were applied to analyse the protein expression patterns, which were indicated by relative grey value of the corresponding protein bands and of the internal reference protein bands.

### 2.10 Reverse transcription quantitative polymerase chain reaction (RT‐qPCR)

RNA extraction kits (AM1552, Thermo Fisher Scientific) were used to extract the total RNA content from the cells and tissues and RNA content from EVs following the instructions, and the RNA concentration was determined. In accordance with the instructions of the miRNA reverse transcription kit (XY001180, Shanghai Xinyuan Ruimin Bioengineering Co., Ltd., Shanghai, China) and complementary DNA (cDNA) reverse transcription kit (XY018011, Xingyuan Ruimin Bioengineering Co., Ltd., Shanghai, China), the cDNA was obtained, and then, the gene fragments were amplified. miR‐150‐5p reverse transcription primers were purchased from Ribobio (Guangzhou, China), while the other target gene primers were synthesized by Takara (Dalian, China) (Table 1). The detection of target gene expression was performed with a fluorescence quantitative PCR instrument (ABI ViiA 7, Daan International Holdings Limited, Guangzhou, China). With GAPDH and U6 serving as the internal parameters, the relative expression of the target gene was calculated using the relative quantitative method (2^−△△^CT method).

**Table 1:** Primer sequences for RT‐qPCR

Gene	Primer sequences (5'‐3')
miR‐150‐5p (Mouse)	F:ACACTCCAGCTGGGTCTCCCAACCCTTGTA
	R:TGGTGTCGTGGAGTCG
CXCL1 (Mouse)	F:ACTGCACCCAAACCGAAGTC
	R:TGGGGACACCTTTTAGCATCTT
U6 (Mouse)	F:CTCGCTTCGGCAGCACA
	R:AACGCTTCACGAATTTGCGT
GAPDH (Mouse)	F:AGGTCGGTGTGAACGGATTTG
	R:TGTAGACCATGTAGTTGAGGTCA

Abbreviations: CXCL1, CXC chemokine‐ligand‐1; GAPDH, glyceraldehyde‐3‐phosphate dehydrogenase; miR‐150‐5p, microRNA‐150‐5p; RT‐qPCR, reverse transcription quantitative polymerase chain reaction.

### 2.11 Dual luciferase reporter gene assay

The target genes of CXCL1 and miR‐150‐5p were predicted using the Starbase website (http://starbase.sysu.edu.cn/agocliprna.php? Source = mRNA) and RNA22 website (https://cm.jefferson.edu/rna22/precomputed/), and validated with the help of a dual luciferase reporter gene assay. The target gene CXCL1 dual luciferase reporter gene vector and mutation at the miR‐150‐5p binding site, namely pGL3‐CXCL1 wild‐type (WT) and pGL3‐CXCL1 mutant type (Mut) were constructed, respectively. These two report plasmids were co‐infected with miR‐150‐5p and pRL‐TK (internal reference plasmid for Renilla luciferase) into the HEK293 cells, respectively. After 48 hours of infection, the cells were lysed and the supernatant was collected according to the procedures provided on the TransDetect Double‐Luciferase Reporter Assay Kit (FR201‐01, TransGen Biotech, Beijing, China). Luciferase activity was detected using a Dual‐Luciferase® Reporter Assay System (E1910, Promega, Madison, WI, USA). The relative luciferase activity was presented by the ratio between firefly luciferase and Renilla luciferase.

### 2.12 Liver injury induced by CCl_4_ administration

A total of 64 mice were randomly classified into control (without any treatment), HF mice (CCl_4_ induced HF mice), CCl_4_ + PBS (HF mice injected with PBS), CCl_4_ + miR‐NC‐EVs (HF mice treated with EVs containing lentiviral plasmid miR‐NC), CCl_4_ + miR‐150‐5p‐EVs (HF mice injected with EVs containing lentiviral plasmid miR‐150‐5p), CCl_4_ + oe‐NC (HF mice injected with lentiviral plasmid oe‐NC), CCl_4_ + miR‐150‐5p‐EVs + oe‐NC (HF mice injected with EVs containing lentiviral plasmid miR‐150‐5p + oe‐CXCL1) and CCl_4_ + miR‐150‐5p‐EVs + oe‐CXCL1 (HF mice injected with EVs containing lentiviral plasmid miR‐150‐5p + oe‐CXCL1) groups (n = 8/group). Briefly, HF mice underwent gavage administration with a single dose of CCl_4_ (3% vol/vol in olive oil) at a dosage of 0.05 mL/kg body weight, twice a week for 8 weeks, while the control mice were given the same amounts of olive oil for 8 weeks. The mice were treated with or without EVs or lentiviral plasmid oe‐CXCL1 for 8 weeks. The EVs (0.4 μg/μL, 100 μL) were subsequently injected aseptically into the mice twice a week for 8 weeks. Forty‐eight hours before CCl_4_ injection, mice in each group were injected with related lentivirus *via* the tail vein using high‐pressure, and the recombinant lentivirus (7.6 × 10^7^ IFU per mouse) was injected into mice at a concentration of 10 mg/kg ^24^, ^25^ After CCl_4_ treatment, the mice were euthanized, and their livers and serums were collected for further analysis.

### 2.13 Enzyme‐linked immunosorbent assay

The serum was centrifuged at 3000 r/minute, and the levels of interleukin‐17 (IL‐17; ab79056, Abcam, mouse), interleukin‐6 (IL‐6; ab100772, Abcam, mouse) and tumour necrosis factor alpha (TNF‐α; ab100785, Abcam, mouse) were detected following the protocols of the ELISA antibody detection kit.

### 2.14 Detection of aspartate transaminase, alanine transaminase and total bilirubin

To assess hepatic injury and function, commercially available kits and a semi‐automatic photometer 5010 (Robert Riele GmbH & Co Kg, Berlin, Germany) were adopted to measure the serum levels of AST (LM‐ALT‐Mu, Lianmai Bioengineering Co., Ltd., Shanghai, China), ALT (LM‐ALT‐Mu, Lianmai) and TB (LM‐12141‐Es, Lianmai).

### 2.15 Haematoxylin‐eosin staining

The liver tissues were fixed in 10% neutral polyformaldehyde (G2131, Solarbio Technology Co., Ltd., Beijing, China) for about 24 hours, dehydrated with graded ethanol, paraffin‐embedded and sliced into 4 μm sections. The prepared tissue sections were transferred to slides, dewaxed in xylene (GS‐RY1215‐12, Guduo) and then rehydrated with gradient ethanol. Next, 100 μL haematoxylin (PT01, Shanghai Bogoo Biotechnology Co., Ltd., Shanghai, China) and eosin (G1424, Solarbio) were applied to stain the sections for 10 minutes or 3 minutes, respectively. The sections were then observed and photographed under a light microscope, and finally reviewed by an experienced pathologist.

The corresponding scores of the severity of pathological changes were recorded: 0, no; 1, mild; 2, moderate; 3, severe; 4, extremely severe; and 5, extremely severe, based on the structural integrity of liver lobules, fatty degeneration or necrosis of liver cells, infiltration of inflammatory cells and hyperplasia of fibrous tissues.

### 2.16 Masson staining

Paraffin sections were obtained from the liver tissues of mice, and the degree of HF was detected following the protocols of the Masson staining kit (G1340, Solarbio). The results were identified as positive if basement membrane and collagen fibres were stained blue or green, immune complex was stained red, and nucleus was stained blue brown. Next, 5 visual fields were randomly observed in each section under a polarized light microscope, and the Image Pro Plus 5.1 image analysis software (Cybernets, USA) was adopted for image analysis. Collagen volume fraction (CVF) was calculated as follows: CVF (%) = collagen area/ full field area × 100%.

### 2.17 Immunohistochemistry

Paraffin sections (4 μm in thickness) were dewaxed with water and subjected to immunohistochemistry. The primary rabbit antibody to collagen I (ab34710, dilution ratio of 1:200), vimentin (ab92547, dilution ratio of 1:200) and secondary antibody to IgG (ab150083, dilution ratio of 1:100) were all purchased from Abcam Inc with the non‐specific normal IgG serving as the NC. Criteria of staining results were as follows: under 200‐ or 400‐times microscope, 5 pathological areas were randomly selected and the number of positive cells in the cells was counted. The density of positive cells can also be classified semi‐quantitatively according to the percentage of positive cells: the number of positive cells < 15% was regarded as negative 0, 15% ~ 25% as (+), 25% ~ 50% as (+ +), 50% ~ 75% as (+ + +) and > 75% as (+ + + +). The results of the experiment were reviewed by an experienced pathologist.

### 2.18 Statistical analysis

All data, representative of three independent experiments in triplicate, are shown as mean ± standard deviation and analysed with the SPSS 21.0 software (IBM, Armonk, NY, USA). Independent sample t test was applied for comparing data between two groups, and one‐way analysis of variance (ANOVA) combined with Tukey's post hoc test was utilized for data comparison among multiple groups. Data comparison among groups at different time‐points was conducted by repeated measures ANOVA, followed by Bonferroni post hoc test. A value of *P* < .05 was indicative of statistical significance.

## 3 RESULTS

### 3.1 miR‐150‐5p was poorly expressed and CXCL1 was highly expressed in HF

**Figure 1:**
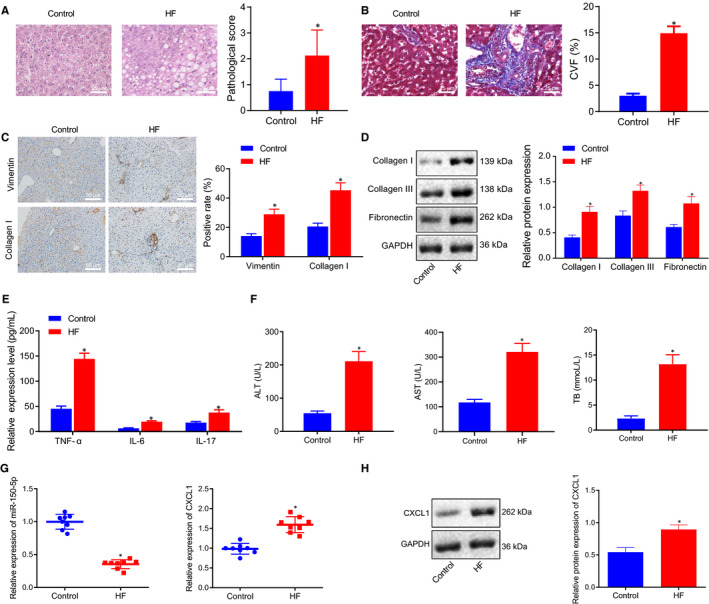
miR‐150‐5p is poorly while CXCL1 is highly expressed in HF. A, The pathological changes of liver tissues of HF and control mice as detected by HE staining (× 400, scale bar = 25 μm). B, The degree of liver fibrosis of HF and control mice as measured by Masson staining (× 400, scale bar = 25 μm). C, The positive expression of vimentin and collagen I in liver tissues of HF and control mice as identified by immunohistochemistry (× 200, scale bar = 50 μm). D, The protein expression of fibrosis‐related factors (collagen I, collagen III and fibronectin) in liver tissues of HF and control mice as determined by Western blot assay. E, The expression of inflammatory factors (TNF‐α, IL‐6, and IL‐17) in serum of HF and control mice as detected by ELISA. F, The expression of liver injury‐related factors (ALT, AST and TB) as measured using commercially available kits. G, The expression of miR‐150‐5p and CXCL1 in liver tissues of HF and control mice as determined by RT‐qPCR. H, The protein expression of CXCL1 in liver tissues of HF and control mice as determined by Western blot assay. *P < .05 *vs*. control. Independent sample t test was used for comparing data between two groups. n = 8

Initially, mice were injected with CCl_4_ to establish HF mouse models. HE and Masson staining were subsequently used to observe the pathological changes and degree of HF, which revealed that the pathological score and CVF of HF mice were significantly higher than those of control mice (Figure 1A,B). The protein expression patterns of vimentin and collagen I in liver tissue were also determined using immunohistochemistry, and a significant elevation in vimentin and collagen I levels were noted in HF mice compared with control mice (Figure 1C). Meanwhile, Western blot assay demonstrated that the protein expression levels of fibrosis‐related factors collagen I, collagen III and fibronectin in HF mice were all notably higher than those in the control mice (Figure 1D). Moreover, ELISA results illustrated that the expression levels of TNF‐α, IL‐6 and IL‐17 as well as ALT, AST and TB levels were markedly higher in HF mice relative to control mice (Figure 1E,F). These findings verified the successful establishment of HF mouse models.

Subsequently, the expression patterns of miR‐150‐5p and CXCL1 were determined with the help of RT‐qPCR and Western blot assay, which revealed that HF mice presented with suppressed miR‐150‐5p and elevated CXCL1 levels compared with the control mice (Figure 1G,H).

### 3.2 Characterization of ADMSCs and EVs

**Figure 2:**
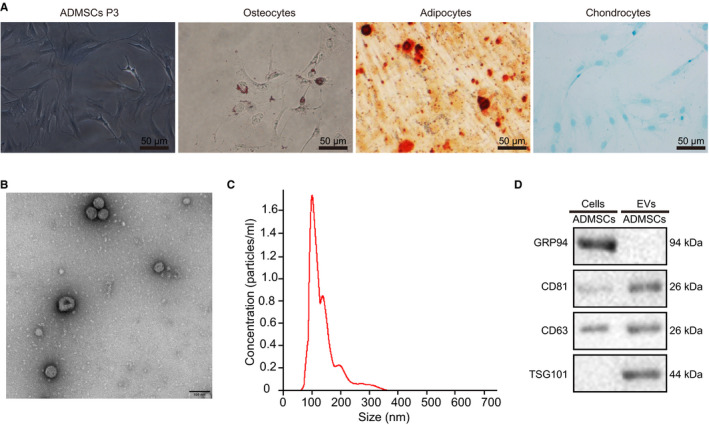
Identification of ADMSCs and EVs. A, The morphology of the third generation of ADMSCs observed under an optical microscope and the staining results of osteoblasts, adipocytes and chondrocytes after differentiation (scale bar = 50 μm). B, The structure of EVs as observed by transmission electron microscopy (scale bar = 100 nm). C, The size and number of EVs as measured through nanoparticle tracking analysis. D, The protein expression of TSG101, CD63, CD81 and GRP94 in cells and EVs as detected by Western blot assay

The third generation of ADMSCs was used to prepare a suspension with a concentration of 1 × 10^6^ cells/mL. Flow cytometry was then carried out to detect the surface antigen phenotype of ADMSCs and non‐ADMSCs. CD166, CD73, CD90, CD44, CD105 and CD29 presented with positive expression, while CD34, CD45, CD14, CD16 and HLA‐DR were negative. In addition, the growth morphology of the third generation of ADMSCs and the osteogenic, lipogenic and chondrogenic differentiation abilities of ADMSCs were observed under a light microscope. These cells were spindle‐shape showing vortex‐shaped or parallel arrangement growth and the nuclei were observed in the middle, most of which were one nucleolus, distributed radially around a point. In addition, ADMSCs differentiated into adipocytes, osteoblasts or chondrocytes after adipogenic differentiation induction, osteogenic differentiation induction or chondrogenic differentiation induction, respectively (Figure 2A).

Afterwards, ADMSCs‐EVs were isolated from the HF modelled mice for subsequent experiments. The morphology of the EVs was observed with the help of transmission electron microscopy, which revealed that most of the EVs were round or oval‐shaped with different sizes, with a diameter of 30‐150 nm (Figure 2B). Nanoparticle tracking analysis was further performed to detect the diameter and number of EVs, which demonstrated that most of the obtained EVs were distributed at 30‐200 nm (Figure 2C). In addition, Western blot assay was employed to determine the expression patterns of specific marker proteins for EVs (CD63, CD81 and TSG101) and endoplasmic reticulum marker (GRP94). The protein expression levels of TSG101, CD63 and CD81 were found to be significantly up‐regulated in the EVs compared with the control cells, while the GRP94 protein was almost not expressed (Figure 2D), indicating that ADMSCs‐EVs were successfully isolated.

### 3.3 ADMSCs‐EVs transferred miR‐150‐5p to HSCs

**Figure 3:**
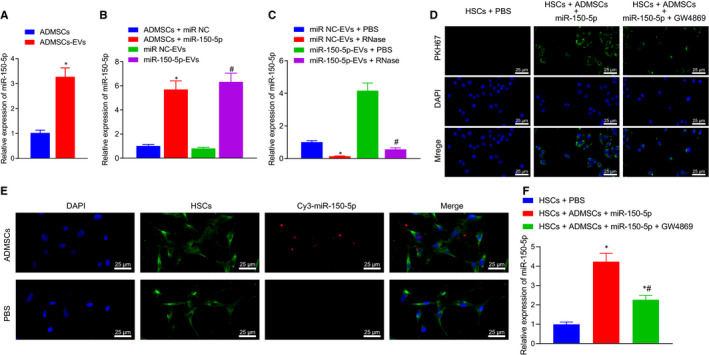
ADMSCs‐EVs transfers miR‐150‐5p to HSCs. A, The expression of miR‐150‐5p in ADMSCs and their corresponding EVs. B, The expression of miR‐150‐5p in ADMSCs and their corresponding EVs after up‐regulation of miR‐150‐5p, detected by RT‐qPCR. C, The expression of miR‐150‐5p in EVs after interfering with the expression of RNA. D, Uptake of ADMSCs containing miR‐150‐5p by HSCs. The nuclei show blue fluorescence, and the EVs show green fluorescence after PKH67 labelling (× 400, scale bar = 25 μm). E, The Cy3‐labelled miR‐150‐5p expression in co‐cultured HSCs as observed under the fluorescence microscope, Cy3 shows red fluorescence, and cell membranes show red fluorescence (× 400, scale bar = 25 μm). F, The expression of miR‐150‐5p in HSCs as detected by RT‐qPCR. *P < .05 *vs*. ADMSCs + miR‐NC, miR‐NC‐EVs + PBS or HSCs + PBS; ^#^ *P* < .05 *vs*. miR‐NC‐EVs, miR‐150‐5p‐EVs + PBS or HSCs + ADMSCs +miR‐150‐5p. One‐way ANOVA combined with Tukey's post hoc test was utilized for data comparison among multiple groups

The miR‐150‐5p expression patterns in ADMSCs and ADMSCs‐EVs separated from control and HF mice were assessed by RT‐qPCR, which illustrated that ADMSCs and ADMSCs‐EVs presented with a markedly increased levels of miR‐150‐5p in comparison with those in control‐ADMSCs or control‐ADMSCs‐EVs (Figure 3A). Subsequently, ADMSCs were infected with lentiviral plasmids oe‐miR‐150‐5p and miR‐NC, and their EVs (miR‐150‐5p‐EVs and miR‐NC‐EVs) were isolated. RT‐qPCR revealed that after treatment of miR‐150‐5p‐mimic, the expression levels of miR‐150‐5p increased in ADMSCs and their secreted EVs (Figure 3B). Furthermore, PBS or RNase was added to miR‐150‐5p‐EVs or miR‐NC‐EVs, respectively, to verify the stability of miR‐150‐5p. Additional RT‐qPCR displayed that the expression of miR‐150‐5p were significantly decreased when the EV membrane was damaged by lysate and affected by RNase (Figure 3C).

Additionally, in order to investigate whether ADMSCs‐EVs containing miR‐150‐5p could be delivered to HSCs, PKH‐67 was adopted to label the ADMSCs‐miR‐150‐5p‐EVs in three treatment groups (HSCs + PBS, HSCs + ADMSCs +miR‐150‐5p, HSCs + ADMSCs +miR‐150‐5p + GW4869). Subsequently, the uptake of ADMSCs‐miR‐150‐5p‐EVs by HSCs was examined under a fluorescence microscope. No green fluorescence was observed in HSCs treated with PBS, while strong green fluorescence was noted in HSCs treated with ADMSCs‐miR‐150‐5p‐EVs, and weak green fluorescence in HSCs treated with ADMSCs‐miR‐150‐5p‐EVs + GW4869, suggesting that ADMSCs‐miR‐150‐5p‐EVs were successfully internalized into the HSCs (Figure 3D). After the ADMSCs overexpressing miR‐150‐5p were labelled with Cy3, the expression patterns of miR‐150‐5p in each group were observed by fluorescence microscopy. It was found that HSCs treated with PBS did not exhibit red fluorescence, while HSCs treated with ADMSCs‐miR‐150‐5p‐EVs showed red fluorescence (Figure 3E). Finally, RT‐qPCR was performed to measure the expression patterns of miR‐150‐5p in the co‐cultured HSCs. The lowest expression levels of miR‐150‐5p were observed in the HSCs treated with PBS. Compared with that in the HSCs treated with ADMSCs‐miR‐150‐5p‐EVs, the expression levels of miR‐150‐5p in HSCs treated with ADMSCs‐miR‐150‐5p‐EVs + GW4869 were significantly lower (Figure 3F). These findings indicated that ADMSCs‐EVs could deliver miR‐150‐5p to HSCs and promote the expression of miR‐150‐5p in HSCs.

### 3.4 miR‐150‐5p delivered by ADMSCs‐EVs alleviated transforming growth factor β‐induced HSC activation

**Figure 4:**
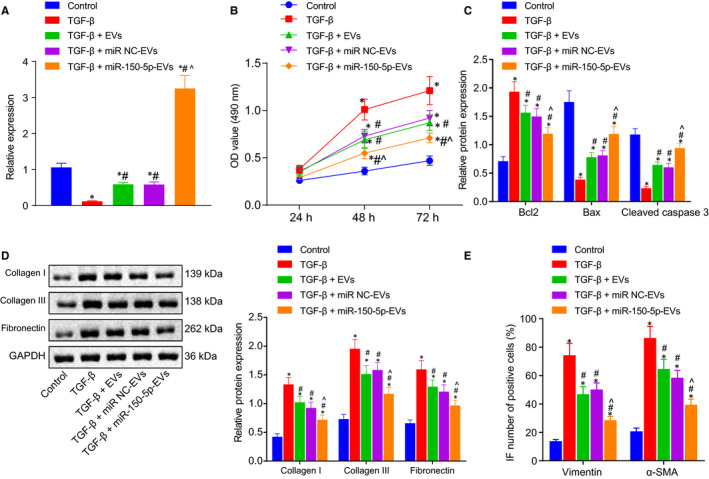
miR‐150‐5p delivered by ADMSCs‐EVs alleviates TGF‐β induced HSC activation. A, The miR‐150‐5p expression in HSCs with different treatment. B, The proliferation of HSCs with different treatment as detected by MTT assay. C, The protein expression of apoptosis‐related factors (Bax, Bcl‐2 and cleaved caspase‐3) with different treatment as determined by Western blot assay. D, The protein expression of fibrosis‐related factors (collagen I, collagen III and fibronectin) with different treatment as determined by Western blot assay. E, Immunofluorescence analysis of the expression of HSC activation markers (vimentin and α‐SMA). *P < .05 *vs*. control. #*P* < .05 *vs*. TGF‐β. ^*P* < .05 *vs*. TGF‐β + miR‐NC‐EVs. NS means *P* > .05. One‐way ANOVA combined with Tukey's post hoc test was utilized for data comparison among multiple groups

To investigate how miR‐150‐5p delivered by ADMSCs‐EVs affects the development of HSCs, TGF‐β (4 ng/mL) was used to induce HSC activation^26^ and followed by co‐culture with ADMSCs‐EVs, miR‐NC‐EVs and miR‐150‐5p‐EVs. According to the results of RT‐qPCR, relative to control HSCs, TGF‐β‐induced HSCs exhibited a striking reduction in the expression levels of miR‐150‐5p. Meanwhile, TGF‐β + EVs, TGF‐β + miR‐NC‐EVs or TGF‐β + miR‐150‐5p‐EVs treatments brought about elevated expression levels of miR‐150‐5p, and compared with TGF‐β + miR‐NC‐EVs treatment, TGF‐β + miR‐150‐5p‐EVs treatment led to the most significant elevation in miR‐150‐5p expression levels (Figure 4A). In addition, MTT assay demonstrated that TGF‐β + EVs, TGF‐β + miR‐NC‐EVs or TGF‐β + miR‐150‐5p‐EVs treatment significantly inhibited cell proliferation, and TGF‐β + miR‐150‐5p‐EVs treatment brought about the most significant inhibition of cell proliferation, whereas miR‐150‐5p‐EVs inhibited TGF‐β‐induced cell proliferation (Figure 4B). Western blot assay further illustrated that the expression levels of Bcl‐2, collagen I, collagen III and fibronectin were increased remarkably, while those of Bax and cleaved caspase‐3 were reduced after TGF‐β induction in HSCs; Bcl‐2, collagen Ⅰ, collagen Ⅲ and fibronectin expressions were notably reduced, while those of Bax and cleaved caspase‐3 were increased after TGF‐β + EVs, TGF‐β + miR‐NC‐EVs or TGF‐β + miR‐150‐5p‐EVs treatment and TGF‐β + miR‐150‐5p‐EVs treatment brought about more significant reduction in Bcl‐2 collagen Ⅰ, collagen Ⅲ and fibronectin expression levels, and increased those of Bax and cleaved caspase‐3 levels (Figure 4C,D). Lastly, immunofluorescence revealed that after TGF‐β induction, HSCs showed elevated expression levels of vimentin and α‐SMA; TGF‐β + EVs, TGF‐β + miR‐NC‐EVs or TGF‐β + miR‐150‐5p‐EVs treatment reduced the vimentin and α‐SMA expression, and compared with TGF‐β + miR‐NC‐EVs, TGF‐β + miR‐150‐5p‐EVs treatment induced more pronounced reduction in the expression levels of vimentin and α‐SMA (Figure 4E). These results showed that EV‐derived miR‐150‐5p could inhibit the TGF‐β‐induced HSC activation.

### 3.5 miR‐150‐5p delivered by ADMSCs‐EVs alleviated HF in vivo

**Figure 5:**
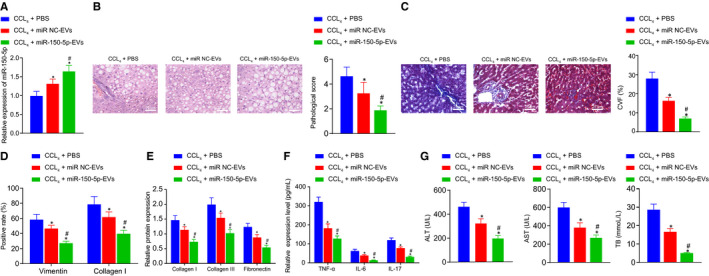
miR‐150‐5p delivered by ADMSCs‐EVs alleviates HF in vivo. A, The expression of miR‐150‐5p in liver tissues of mice with different treatment as determined by RT‐qPCR. B, The pathological changes of liver tissues of mice with different treatment as identified by HE staining (× 400, scale bar = 25 μm). C, The degree of fibrosis in liver tissues of mice with different treatment as identified by Masson staining (× 400, scale bar = 25 μm). D, The positive expression of vimentin and collagen I in liver tissues of mice with different treatment as determined by immunohistochemistry. E, The protein expression of fibrosis‐related factors (collagen I, collagen III and fibronectin) in liver tissues of mice with different treatment as detected by Western blot assay. F, The expression of inflammatory factors (TNF‐α, IL‐6 and IL‐17) in serum of mice with different treatment as measured by ELISA, G, The expression of liver injury‐related factors (ALT, AST and TB) in mice with different treatment as detected using commercially available kits. *P < .05 *vs*. CCL_4_ + PBS; #*P* < .05 *vs*. CCL_4_ + miR‐NC‐EVs. Data among multiple groups were compared by one‐way ANOVA combined with Tukey's post hoc tests. n = 8

Furthermore, to study the effect of miR‐150‐5p delivered by ADMSCs‐EVs on HF in vivo, PBS, miR‐NC‐EVs and miR‐150‐5p‐EVs were injected into the CCl_4_‐induced HF modelled mice. RT‐qPCR results displayed that the lowest expression of miR‐150‐5p was found in the presence of PBS, and compared with miR‐NC‐EVs, miR‐150‐5p‐EVs injection restored the miR‐150‐5p expression (Figure 5A). HE and Masson staining were also conducted to observe the pathological changes and fibrosis degree of liver tissues, which revealed that compared with PBS, miR‐NC‐EVs and miR‐150‐5p‐EVs contributed to decreased pathological scores and CVF. Relative to miR‐NC‐EVs, miR‐150‐5p‐EVs treatment brought about a reduction in the pathological scores and CVF (Figure 5B,C). In addition, immunohistochemistry demonstrated that the protein expression levels of vimentin and collagen I were lower upon miR‐NC‐EVs or miR‐150‐5p‐EVs treatment compared with PBS and that compared with miR‐NC‐EVs, miR‐150‐5p‐EVs treatment inhibited the protein expressions of vimentin and collagen I (Figure 5D). As illustrated in Western blot assay, the expression levels of CXCL1, collagen I, collagen III and fibronectin were decreased by miR‐NC‐EVs and miR‐150‐5p‐EVs compared with the PBS treatment, while miR‐150‐5p‐EVs injection brought about a significant inhibition in CXCL1, collagen I, collagen III and fibronectin expressions (Figure 5E). Furthermore, ELISA revealed that the expression levels of TNF‐α, IL‐6 and IL‐17 were lower in the serum of HF mice injected with miR‐NC‐EVs and miR‐150‐5p‐EVs compared with those injected with PBS. In comparison with miR‐NC‐EVs, miR‐150‐5p‐EVs caused a decrease in the expressions of TNF‐a, IL‐6 and IL‐17 (Figure 5F). Lastly, it was found that miR‐NC‐EVs and miR‐150‐5p‐EVs contributed to decreased expression levels of ALT, AST and TB when compared with PBS. Relative to miR‐NC‐EVs, miR‐150‐5p‐EVs diminished the expressions of ALT, AST and TB (Figure 5G). The above results suggested that miR‐150‐5p delivered by ADMSCs‐EVs is capable of alleviating HF in vivo.

### 3.6 miR‐150‐5p down‐regulated CXCL1

**Figure 6:**
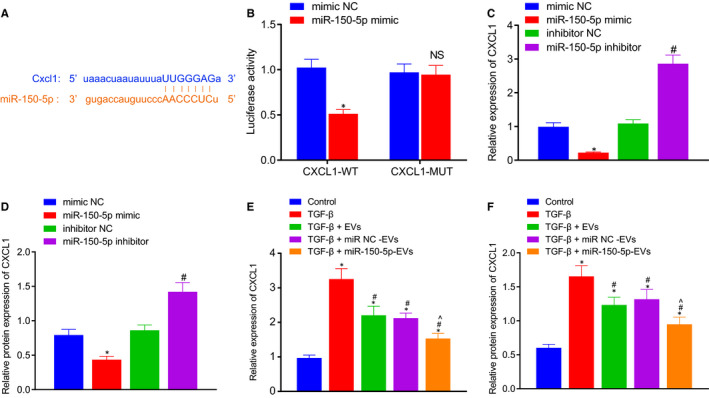
miR‐150‐5p targets and down‐regulates CXCL1. A, The target‐binding site between miR‐150‐5p and CXCL1 3'UTR as predicted on the Targetscan website. B, Dual luciferase reporter gene assay verifying that miR‐150‐5p could target to CXCL1. C, The mRNA expression of CXCL1 in HSCs with different treatment as determined by RT‐qPCR. D, The protein expression of CXCL1 in HSCs with different treatment as determined by Western blot assay. E, The mRNA expression of CXCL1 in HSCs in co‐culture system as determined by RT‐qPCR. F, The protein expression of CXCL1 in HSCs in co‐culture system as determined by Western blot assay. *P < .05 *vs*. mimic‐NC or inhibitor‐NC or control. #*P* < .05 *vs*. TGF‐β. ^*P* < .05 *vs*. TGF‐β + miR‐NC‐EVs. NS means *P* > .05. Independent sample *t* test was used for comparing data between two groups, and one‐way ANOVA combined with Tukey's post hoc test was utilized for data comparison among multiple groups

The TargetScan website (http://www.targetscan.org/vert71/) was initially retrieved to predict the downstream target gene of miR‐150‐5p, which revealed the presence of a targeting binding site between miR‐150‐5p and CXCL1 (Figure 6A). Subsequently, dual luciferase reporter gene assay was carried out to verify the targeting relationship between miR‐150‐5p and CXCL1. After site‐directed mutation of 3’‐UTR region of CXCL1 mRNA, the luciferase signal in response to CXCL1‐WT/miR‐150‐5p mimic co‐transfection was found to be markedly decreased, indicating that miR‐150‐5p could specifically bind to CXCL1 (Figure 6B). In addition, HSCs were then infected with miR‐150‐5p mimic and its inhibitor. As shown in RT‐qPCR and Western blot assay, miR‐150‐5p mimic inhibited the CXCL1 expression, while the miR‐150‐5p inhibitor brought about the opposite effect (Figure 6C,D). After HSCs were co‐cultured with ADMSCs‐EVs, miR‐NC‐EVs and miR‐150‐5p‐EVs, respectively, the related RNA and total protein contents were extracted. RT‐qPCR and Western blot assay illustrated that the expression levels of CXCL1 were markedly increased in TGF‐β‐induced HSCs compared with those in control HSCs. The expression of CXCL1 was found to be reduced by TGF‐β + EVs, TGF‐β + miR‐NC‐EVs or TGF‐β + miR‐150‐5p‐EVs treatment, and TGF‐β + miR‐150‐5p‐EVs treatment brought about the most significant inhibition of CXCL1 expression compared with TGF‐β + miR‐NC‐EVs (Figure 6E,F). Overall, these findings indicated that ADMSCs‐EVs‐derived miR‐150‐5p could inhibit the expression of CXCL1.

### 3.7 ADMSCs‐EVs containing miR‐150‐5p mediated CXCL1 inhibited TGF‐β‐induced HSC activation

**Figure 7:**
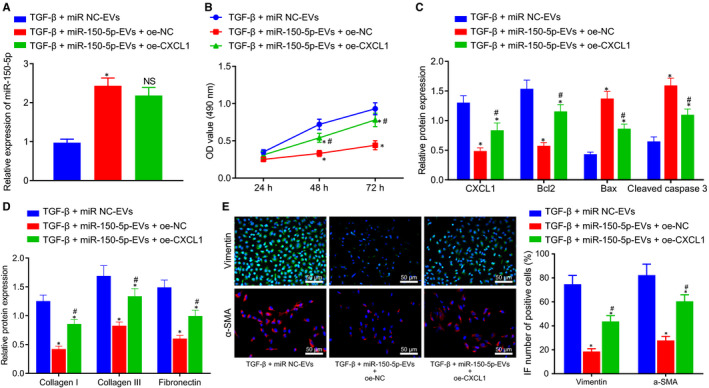
ADMSCs‐EVs containing miR‐150‐5p mediates CXCL1 to inhibit TGF‐β‐induced HSC activation. A, The expression of miR‐150‐5p in HSCs in co‐culture system as determined by RT‐qPCR. B, The proliferation of HSCs in co‐culture system as examined by MTT assay. C, The expression of CXCL1 and apoptosis‐related factors (Bax, Bcl‐2 and caspase‐3) in HSCs in co‐culture system as determined by RT‐qPCR. D, The protein expression of fibrosis‐related factors (collagen Ⅰ, collagen Ⅲ and fibronectin) in HSCs in co‐culture system as determined by Western blot assay. E, The expression of HSC activation markers (vimentin and α‐SMA) in HSCs in co‐culture system as determined by immunofluorescence. vimentin shows green fluorescence, α‐SMA shows red fluorescence (× 200, scale bar = 50 μm). *P < .05 *vs*. TGF‐β + miR‐NC‐EVs. #*P* < .05 *vs*. TGF‐β + miR‐150‐5p‐EVs + oe‐NC. NS means *P* > .05. Data among multiple groups were compared by one‐way ANOVA combined with Tukey's post hoc tests

To elucidate the effect of ADMSCs‐EVs‐derived miR‐150‐5p on the activation of HSCs by regulating the expression of CXCL1, TGF‐β was used to induce HSC activation, followed by treatment with miR‐NC‐EVs, miR‐150‐5p‐EVs + oe‐NC and miR‐150‐5p‐EVs + oe‐CXCL1. RT‐qPCR results revealed that when compared to miR‐NC‐EVs, miR‐150‐5p‐EVs markedly increased the miR‐150‐5p expression, while oe‐CXCL1 did not alter the miR‐150‐5p expression compared with oe‐NC (Figure 7A). In addition, MTT assay demonstrated that relative to miR‐NC‐EVs, miR‐150‐5p‐EVs led to inhibited HSC proliferation, while CXCL1 over‐expression reversed this trend (Figure 7B). Western blot assay further displayed that miR‐150‐5p‐EVs brought about a decline in the expression levels of CXCL1 and Bcl‐2, and increased those of Bax and cleaved caspase‐3, while oe‐CXCL1 treatment could reverse these trends (Figure 7C). Western blot assay on the protein expression patterns of fibrosis‐related factors displayed that the expression levels of collagen I, collagen III and fibronectin were inhibited in response to miR‐150‐5p‐EVs than that upon miR‐NC‐EVs, but the trend could be reversed by oe‐CXCL1 treatment (Figure 7D). Finally, immunofluorescence revealed that miR‐150‐5p‐EVs inhibited the expression levels of vimentin and α‐SMA compared with miR‐NC‐EVs, relative to miR‐150‐5p‐EVs + oe‐NC, and CXCL1 over‐expression could abrogate this trend (Figure 7E). Collectively, these findings indicated that CXCL1 expression was inhibited by miR‐150‐5p in the ADMSCs, which prevented the activation of HSCs induced by TGF‐β.

### 3.8 ADMSCs‐EVs containing miR‐150‐5p inhibited CXCL1 to alleviate HF in vivo

**Figure 8:**
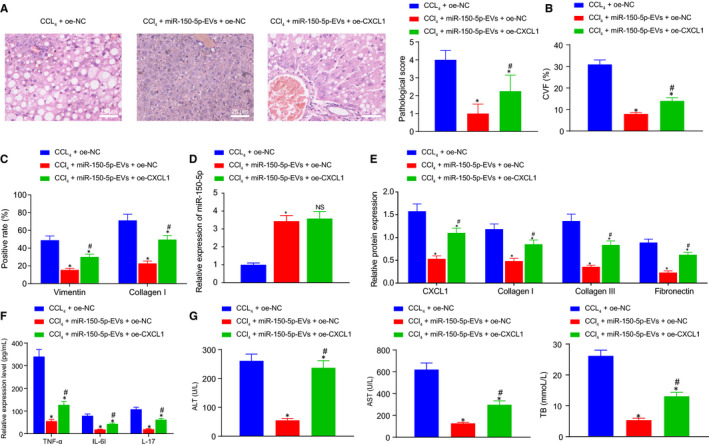
ADMSCs‐EVs containing miR‐150‐5p mediates CXCL1 to alleviate HF in vivo. A, The pathological changes of liver tissues of HF mice with different treatment as detected by HE staining (× 400, scale bar = 25 μm). B, The degree of liver fibrosis of HF mice with different treatment as measured by Masson staining. C, The positive expression of vimentin and collagen I in liver tissues of HF mice with different treatment as identified by immunohistochemistry. D, The expression of miR‐150‐5p in liver tissues of HF mice with different treatment as determined by RT‐qPCR. E, The protein expression of fibrosis‐related factors (collagen I, collagen III and fibronectin) in liver tissues of HF mice with different treatment as determined by Western blot assay. F, The expression of inflammatory factors (TNF‐α, IL‐6 and IL‐17) in serum of HF mice with different treatment as detected by ELISA. G, The expression of liver injury‐related factors (ALT, AST and TB) as measured using commercially available kits. *P < .05 *vs*. CCL_4_ + oe‐NC. # *P* < .05 *vs*. CCL_4_ + miR‐150‐5p‐EVs + oe‐NC. NS means *P* > .05. Data among multiple groups were compared by one‐way ANOVA combined with Tukey's post hoc tests. n = 8

Lastly, in vivo experimentation was carried out to explore the regulatory role of ADMSCs‐EVs containing miR‐150‐5p in HF in vivo through regulating CXCL1. HF mice were injected with oe‐NC, miR‐150‐5p‐EVs + oe‐NC, miR‐150‐5p‐EVs + oe‐CXCL1. Subsequent HE and Masson staining illustrated that compared with oe‐NC, miR‐150‐5p‐EVs reduced the pathological scores and CVF in HF mice, which were reversed by oe‐CXCL1 treatment (Figure 8A,B). As expected, immunohistochemistry showed that in HF mice, versus oe‐NC, miR‐150‐5p‐EVs reduced the protein expression levels of vimentin and collagen I, and these could be reversed by CXCL1 over‐expression (Figure 8C). Moreover, RT‐qPCR results displayed that in comparison with oe‐NC, miR‐150‐5p‐EVs up‐regulated the expression levels of miR‐150‐5p in liver tissues, but relative to miR‐150‐5p‐EVs + oe‐NC, oe‐CXCL1 injection did not alter the expression of miR‐150‐5p (Figure 8D). Western blot assay revealed that compared with oe‐NC, miR‐150‐5p‐EVs down‐regulated the expression levels of CXCL1, collagen Ⅰ, collagen Ⅲ and fibronectin, while the trend could be abrogated by oe‐CXCL1 treatment (Figure 8E). ELISA results displayed that miR‐150‐5p‐EVs decreased the expression levels of TNF‐α, IL‐6 and IL‐17 versus oe‐NC, while relative to miR‐150‐5p‐EVs + oe‐NC, miR‐150‐5p‐EVs + oe‐CXCL1 injection brought about an increase in the expressions of TNF‐α, IL‐6 and IL‐17 that had been decreased by miR‐150‐5p‐EVs (Figure 8F). Finally, it was found that miR‐150‐5p‐EVs reduced the levels of ALT, AST and TB versus CCl_4_ + oe‐NC, relative to CCl_4_ + miR‐150‐5p‐EVs + oe‐NC, but this trend was also reversed by oe‐CXCL1 treatment (Figure 8G). Taken together, these results demonstrated that EVs derived miR‐150‐5p could inhibit the CXCL1 expression to alleviate HF in vivo.

## 4 DISCUSSION

HF is an intense repair as well as cicatrization mechanism‐related disorder, with its end‐stage cirrhosis accounting for high global morbidity and mortality rates.^27^ It has been highlighted that EVs have the potential for HF therapy through mediation of intercellular miR delivery between HSCs.^9^ In the current study, we aimed to investigate the role of ADMSCs‐derived EVs containing miR‐150‐5p in HF, which uncovered an alleviatory role.

Firstly, we uncovered that miR‐150‐5p was poorly expressed and CXCL1 was highly expressed in HF. Similarly, down‐regulated expression levels of serum miR‐150‐5p have been previously documented in patients with higher HF grading.^28^ More notably, a particular study demonstrated that miR‐150 suppressed HSC activation by down‐regulating HOXA transcript at the distal tip, thereby serving as a potential therapeutic target for HF.^29^ On the other hand, diminished expressions of CXCL1 as a result of deficiency of IL‐17RA were previously found to be capable of mediating cholesterol synthesis, indicating its regulatory role in HF.^30^ Up‐regulation of CXCL1 by CD147 is also known to stimulate HSC activation *via* autocrine, which further implicates CXCL1 with HF development.^31^ All these findings and reports highlight the involvement of miR‐150‐5p and CXCL1 in HF development.

Additionally, our findings revealed that ADMSCs‐EVS could transport miR‐150‐5p to HSCs, which prevented HSC activation. Transplantation of ADMSCs was previously demonstrated to be beneficial for the amelioration of CCl_4_‐induced HF in rat models.^32^ Consistently, ADMSCs have also been found to be effective at alleviating HF through the suppression of HSC activation and proliferation, which is very much in line with our findings.^33^ Meanwhile, studies have illustrated that EVs also possess the ability to suppress HSC activation and consequently alleviate HF.^34^ More importantly, several previous studies have reported that miR‐150 family can be delivered by EVs for intercellular communication, which underscores the mir‐150‐5p‐EV relationship indicated in the current study. One such study unfolded that EVs carried miR‐150‐5p from Tregs to dendritic cells,^35^ whereas EVs have been documented to transport miR‐150 from smooth muscle cells to endothelial cells in other instances.^36^ Furthermore, MSCs‐derived EVs could accelerate the intracellular transfer of miR‐150‐5p between cells, which was highlighted as potential therapeutic strategy against joint destruction in RA.^37^

Further experimentation in our study revealed that ADMSCs‐EVs containing miR‐150‐5p brought about a marked reduction in the levels of fibrosis‐related factors (collagen I, collagen Ⅲ and fibronectin) both in vivo and in vitro, as well as reduced the levels of HSC activation (vimentin and α‐SMA), apoptosis‐related factors (reduced Bcl2, elevated Bax and cleaved caspase‐3) and HSC proliferation in vitro. EVs secreted by miR‐181‐5p‐containing ADMSCs were similarly demonstrated to prevent HF *via* autophagy activation by down‐regulating collagen I, vimentin, α‐SMA and fibronectin levels in livers.^16^ In addition, up‐regulated expressions of apoptotic factors Bax and cleaved caspase3 have also been previously correlated with inhibited activation of HSCs and HF.^38^ Moreover, one particular study reported, ADMSCs‐derived EVs regulated the miR‐122 communication between ADMSCs and HSCs, wherein miR‐122 targeted several genes including insulin‐like growth factor receptor 1, Cyclin G (1) and prolyl‐4‐hydroxylase α1, thereby inhibiting HSC activation and ameliorating collagen deposition to favour the therapeutic efficacy of CCl_4_‐induced HF.^17^ Furthermore, in vivo experiments in our study demonstrated that ADMSCs‐EVs derived miR‐150‐5p inhibited CVF, reduced the levels of inflammatory factors (TNF‐α, IL‐6 and IL‐17), hepatic injury and function‐associated indicators (ALT, AST and TB). Reduced CVF was reported as an indicator of liver injury amelioration,^39^ while TNF‐α and IL‐17 are well‐known pro‐inflammatory cytokines responsible for HF pathogenesis,^40^ and further, the presence of IL‐6 in human fibrotic livers also plays a role in favouring HF development.^41^ Moreover, diminished levels of AST, ALT and TB have also been previously reported to aid in the attenuation of HF.^42^ Previous study unfolded that miR‐150 could target and decrease CXCL1 expression in mice overexpressing or underexpressing Kruppel‐like factor 2.^20^ All these evidences indicate that miR‐150‐5p delivered by ADMSCs‐EVs could target CXCL1, and consequently suppress HF development both in vivo and in vitro.

To summarize, the current study revealed that EVs derived from ADMSCs deliver miR‐150‐5p to down‐regulate the expression of CXCL1, which inhibits the development of HF. Our findings highlight the therapeutic potential of miR‐150‐5p‐containing ADMSCs‐derived EVs for HF treatment, which still requires further validation to improve the quality of life of patients plagued by HF.

## CONFLICT OF INTEREST

The authors declare that there is no conflict of interest.

## AUTHOR CONTRIBUTION

**Zhiyong Du:** Conceptualization (equal); Investigation (equal); Writing‐original draft (equal). **Tianchong Wu:** Data curation (equal); Methodology (equal); Visualization (equal). **Linsen Liu:** Formal analysis (equal); Validation (equal). **Biwei Luo:** Project administration (equal); Supervision (equal). **Cuifeng Wei:** Resources (equal); Software (equal); Writing‐review & editing (equal).

## Data Availability

Research data not shared.
